# The Treatment of Vomiting with Large Doses of Oxalate of Cerium

**Published:** 1885-10-20

**Authors:** W. K. Chittick


### THE TREATMENT OF VOMITING WITH LARGE DOSES OF OXALATE OF CERIUM.

By W. K. Chittick, M. D.

Read before the Detroit Academy of Medicine.

One of the most annoying, sometimes serious and even dangerous complications of disease, is vomiting. All who have had to contend with this annoying trouble in gastric, intestinal and other disorders, can, probably, appreciate the relief that one feels when he can place his hand on a reliable remedy, and one that is not likely to interfere with other treatment when the case is complicated with vomiting. We have such a remedy in oxalate of cerium. Judging from my own experience, which has been considerable, I know of no other remedy that will fulfill all the indications so well as this one. Of course, it will not answer in all cases ; it is not a "cure all," but, more than any other one remedy, it seems to answer the purpose.

This is not a new remedy. It has been used in the treatment of vomiting for some years, especially for the vomiting of pregnancy. It was first introduced for this purpose by Sir J. Y. Simpson, and has been in use, more or less, ever since. Simpson obtained remarkable results with this remedy when everything else failed, and by using only small doses of the drug. Many others have used it since that time, but with indifferent success. The cause of this, I believe, lay in the small doses used.

To obtaining gratifying results with this remedy, it must be used in much larger doses than those laid down in the text-books, or the dose given by Simpson.

That the drug is quite harmless, I have satisfied myself. That it may be given in large doses without any ill effect, seems proven by the results obtained by the use of the drug in those cases in which it was used as a remedy for cough, in phthisical patients, and which were reported to the New York Therapeutical Society, in April, 1880 (N. Y. Medical Record). In those cases, doses of 3, 5, and 10 grains were given two and three times a day, with relief in some cases, and benefit in all. The greatest relief, however, was obtained in those cases in which the cough was caused by or associated with an irritable stomach.

Much has been said of this remedy in the treatment of the vomiting of pregnancy. I have used it in these cases with success, especially when combined with ingluvin. If these combinedremedies fail to control the vomiting in these cases, then the next best thing to do is to dilate the cervix uteri.

Many other remedies are used to control vomiting, and with very good success, under favorable conditions. In one case I used small doses of ipecac alone. It stopped the vomiting, but did it slowly and not completely until nearly twenty-four hours after I began the administration of it. But this was a severe case, and the patient had been vomiting for—her husband said—two or three days before I was called in. I determined to see what ipecac would do, and was satisfied that it gave relief. Opium is a standard remedy, but when I use it I prefer the alkaloid codeia or the preparation called scale opium, which is deprived of the nauseating qualities of the crude drug. For the vomiting of summer diarrhœa in children, very small doses of calomel seem to answer better than anything else, and, if followed by milk digested with extractum pancreatis, will, in the majority of cases, aid the little sufferer to a speedy recovery.

Bismuth, hydrocyanic acid, carbonic acid gas, lime water, bicarbonate of soda, gnaphalium, ice, and small mustard poultices over the pit of the stomach, are remedies that have their value; but above all these, and especially where a sedative action is desirable, stands oxalate of cerium.

My first experience with the use of this drug in large doses in the treatment of vomiting, was gained in the clinical lectures of Dr. George P. Andrews, and, afterwards, in compounding his prescriptions; and, still later, it has been my pleasure to observe the effect of his use of the drug in hospital and private practice, and, so far as I have been able to learn, he is the first to use it in large doses, frequently repeated, for vomiting.

Oxalate of cerium is as harmless, and may be given in as large doses, as bismuth. I have seen it given in scores and scores of cases, and have yet to see the case in which it has done any harm.

When I speak of large doses I mean from 5 to 30 grains, or even more. An effectual dose is from 8 to 10 grains, given every two, three or four hours until relief is obtained. In giving these large doses we obtain relief much more quickly than if we were to give a greater number of small doses. It is best given in powder form or upon the tongue. Sometimes it is desirable to give it combined with other remedies, such as codeia, scale opium, bismuth, ipecac, calomel, ingluvin, pepsin, etc., but this will depend upon the purpose for which it is given. It is our custom to prescribe the remedies that the diseased condition calls for, and, if vomiting occurs, to order the cerium alone, in powders, to be taken as needed. If it is desirable to give but one prescription, then it may be combined with almost any pulverized medicine that is agreeable to the taste; thus: for pain, it may be given with codeia or opium, and, as the former drug is not any stronger in its action than opium itself, it is necessary to give it in fully as large doses; if headache is present, powdered ergot, digitalis, caffein or codeia are useful and efficient drugs to combine with it.

In regard to the physiological action of the drug, I can give you no more information than is contained in the text-books. To study the drug so as to give reliable results, would require investigations that time will not, at present, permit me to make. I presume that most of its action is due to its direct sedative and astringent action on the terminal nerve filaments of the pneumogastric nerve.

The following cases will illustrate the action of the drug:

CASE I.

Mrs. S——; ovaritis; intense pain, and tenderness; had been vomiting for three days before I saw her. She could take no food or drink, but sucked small pieces of ice; even the little water that she got from the ice was rejected. Ordered suppositories for the pain, and oxalate of cerium, in 10-grain doses, to be given every hour until vomiting ceased. She could not swallow the powders without at least two or three teaspoonfuls of water, which so irritated the stomach that they were not retained; I then ordered them in capsules, and to be swallowed without water. They were retained, and patient gradually grew better, and in eighteen hours the vomiting had ceased. This is the first case in which I was obliged to give the cerium in capsules.

CASE II.

Mrs. S——; peritonitis; stercoraceous vomiting; an unpromising case, but one that was greatly relieved by 20-grain doses of cerium, given every two to four hours, with codeine and bismuth.

CASE III.

Mrs. G——; pregnant; always troubled with severe nausea from a very early period. Ordered 10-grain doses, with 10 grains of ingluvin, three times a day. Vomiting checked as long as she took the medicine; when she stopped, it would commence again. She continued the medicine for about a month, when I lost sight of her.

CASE IV.

Mrs. B——; pregnant; began feeling nausea about second month; tried various remedies with little benefit until I prescribed cerium alone in 12-grain doses. This gave very marked relief, which continued as long as she would use the medicine. Twice she stopped it, but found relief on returning to it.

### CASE V.

Mrs. D—— ; uterine trouble ; occasionally troubled with sympathetic vomiting. Eight-grain doses would relieve her in a few hours.

### CASE VI.

Mrs. A—— ; hospital patient ; irritation of the stomach due to excessive use of alcoholic stimulants. A few 10-grain doses relieved her.

Any number of cases like the above case might be reported, but time will not permit.

### CASE VII.

One of the Sisters of St. Mary's Hospital ; cancer of the stomach ; under the care of Dr. Andrews. Oxalate of cerium gave satisfactory relief, and seemed to have some influence over the pain.

### CASE VIII.

Sister Superior at St. Mary's school ; functional dyspepsia at time of menopause ; also under Dr. Andrews' care. Here the cerium gave very marked relief.

### CASE IX.

Miss F—— ; vomiting due to reflex irritation caused by uterine and ovarian trouble. Several 10-grain doses of cerium relieved her.

### CASE X.

Mrs. C—— ; hospital patient ; had been ill for three weeks with malarial fever ; had been vomiting for six days before entering the hospital. Oxalate of cerium in 10-grain doses was ordered, and the next day patient was able to retain food. After this, the patient began to get along nicely, the improvement in the stomach seemed to afford the anti-periodic remedies a better chance of being assimilated. Was kept on cerium, three times a day, for several days.

### CASE XI.

Miss R—— ; hospital patient ; acute tuberculosis ; stomach occasionally becomes affected, but is always relieved by cerium in 8 or 10-grain doses.

### CASE XII.

Clara J—— ; acute tuberculosis, third stage ; diseased condition seems to have extended to stomach ; consequently, there is almost constant disturbance of that organ. Cerium proved to be the best remedy for this patient, and she is obliged to take it every day. While under its influence, she is enabled to get some rest, and to take a little food. It has never caused any disagreeable symptoms, and has proved a great boon to the patient.

Dr. Robert Myers, of Savannah, Ga., has had a large experience in the use of oxalate of cerum in large doses, for stomach and other disorders, and was himself cured of an obstinate dyspepsia by the use of this drug, after other better known remedies had failed.

The doctor related to me the case of an old colored woman who is afflicted with asthma, and on whom the ordinary remedies for this disease failed to give relief. A short time before coming on his visit North, he gave her 30-grain doses of oxalate of cerium with the most happy effect.

To mention more cases would be only to repeat what I have already said. I will say, however, that it has been used in a very large number of cases in the female wards of St. Mary's Hospital, and is looked upon there by the Sisters as *the* remedy to use in the majority of cases of stomach trouble that cause vomiting, and that they use it of their own accord, if the physician is not at hand and the case is urgent.

I submit the foregoing to your consideration, and hope that those of you who have not used it will give it a fair trial.—*Detroit Lancet.*

